# Autologous Osteochondral Transplantation in Large Osteochondral Defects—A Follow-Up of 40 Patients After Talus Re-Surfacing

**DOI:** 10.3390/diagnostics16020351

**Published:** 2026-01-21

**Authors:** Alice Wittig-Draenert, Martin Breitwieser, Patrick Marko, Wolfgang Hitzl, Jürgen Bruns

**Affiliations:** 1 Department for Orthopedic Surgery and Traumatology, Paracelsus Medical University, 5020 Salzburg, Austria; 2 Department of Ophthalmology and Optometry, Paracelsus Medical University Salzburg, 5020 Salzburg, Austria; 3 Research Program Experimental Ophthalmology & Glaucoma Research, Paracelsus Medical University, 5020 Salzburg, Austria; 4 Department for Orthopedic Surgery, Diaconial Hospital Hamburg, 22767 Hamburg, Germany

**Keywords:** autologous osteochondral transplantation, ankle resurfacing, osteochondral lesions of the talus, donor site morbidity, diamond bone cutting system

## Abstract

**Background/Objectives**: Large osteochondral lesions of the talus (OLT) pose a major challenge because their size and depth often exceed the indications for bone marrow stimulation, and durable biological repair remains difficult to achieve. However, evidence for autologous osteochondral transplantation (AOT) in extensive talar defects is still limited. **Methods**: In this retrospective cohort, 40 consecutive patients ≥ 14 years with ICRS grade III–IV lesions of the talar dome were treated with AOT at a tertiary referral center. One to three overlapping cylindrical osteochondral grafts (mean diameter 0.9 cm) were harvested from non-weight-bearing regions of the ipsilateral patellofemoral groove using a water-cooled diamond trephine system and implanted press-fit into the talar dome. Donor sites were refilled with autologous iliac crest bone cylinders and hydroxyapatite substitute. Pain (Numeric Rating Scale, NRS) and function (AOFAS Ankle–Hindfoot Score) were recorded preoperatively and at 3, 6, 9, and 12 months, and changes over time were analyzed using generalized estimating equations. **Results**: Mean defect size was 137.4 ± 31.9 mm^2^, and 82.5% of lesions were ICRS grade III. NRS pain improved from 5.69 ± 2.52 preoperatively to 0.53 ± 0.98 at 12 months (*p* < 0.001). AOFAS score increased from 63.79 ± 2.55 to 97.36 ± 2.49 (*p* < 0.001). Age and graft location significantly influenced postoperative pain, whereas graft size and sex did not. No infections, graft failures, conversions to arthrodesis or arthroplasty, or clinically relevant donor-site symptoms occurred. **Conclusions**: Multi-plug AOT using a diamond trephine system provides substantial and durable pain relief and functional improvement in patients with large OLT, with low complication and donor-site morbidity rates. These findings support AOT as a joint-preserving option for extensive talar defects and justify further prospective, comparative studies with long-term follow-up.

## 1. Introduction

Articular cartilage lesions of the lower limb remain a major challenge in orthopedic surgery. Their limited intrinsic healing capacity and the involvement of both cartilage and subchondral bone make them a relevant risk factor for chronic pain, functional impairment, and early degenerative joint disease [1,2]. Although a wide range of biological and surgical approaches has been proposed, true regeneration of the osteochondral unit is still difficult to achieve, and long-term data remain limited [3,4].

These limitations are particularly evident in the ankle joint, where osteochondral lesions of the talus (OLT) are a common and clinically relevant cause of ankle pain. They involve damage to the talar articular cartilage and its underlying subchondral bone and most often develop after ankle sprains, dislocations, or fractures [5,6]. Non-traumatic fac-tors such as ischemia or metabolic disorders can also contribute to their development, and the talus’ limited vascularity and lack of muscular attachments make spontaneous healing difficult once the osteochondral unit is injured [6,7].

Clinically, patients often present only months after the initial trauma with deep ankle pain during or after weight-bearing activities, sometimes accompanied by stiffness, swelling, or mechanical symptoms. Early examination and imaging findings may resemble soft-tissue injury, and OLTs are therefore frequently overlooked or misdiagnosed [5,8]. This delayed recognition contributes to lesion progression, which is particularly relevant for larger defects—the focus of this study [9,10,11,12].

While smaller, contained lesions without major structural compromise may initially be managed non-operatively through activity modification, immobilization, physiotherapy, or injection therapies, larger or unstable defects often require surgical intervention [13,14]. For focal defects of modest size, bone marrow stimulation (BMS) techniques such as microfracture are widely used and can provide good short- to mid-term outcomes, but with increasing lesion size, depth, or cystic transformation, BMS becomes less suitable [15,16].

The current literature suggests that lesions larger than approximately 100 mm^2^ or 10 mm in diameter, cystic defects, shoulder lesions, and cases after failed primary surgery are better managed with cartilage transplantation techniques [6,17]. These include autologous or allogeneic osteochondral grafting, autologous chondrocyte-based techniques, and periosteum-covered bone grafts. Among these options, autologous osteochondral transplantation (AOT) has become an established procedure for restoring the talar dome with hyaline cartilage and viable subchondral bone taken from non-weight-bearing regions of the knee [18,19].

Several studies have reported favorable clinical outcomes after AOT for talar lesions, including high rates of pain relief, functional improvement, and return to sports [20,21]. At the same time, important questions remain. Donor-site morbidity at the knee, technical difficulty in achieving precise graft congruency in the ankle, and the impact of multiple overlapping plugs in larger defects are still debated [19,21,22]. Prognostic factor analyses suggest that patient-related factors such as age, BMI, and preoperative status influence outcome, whereas lesion size itself is not consistently associated with worse results, but most series include relatively small or heterogeneous defects and short-to-medium follow-up [23,24].

Large osteochondral lesions of the talus, particularly those exceeding 150–200 mm^2^, pose a greater reconstructive challenge and often require resurfacing of a substantial portion of the talar dome. Although AOT has shown favorable results for small-to-moderate OLT, evidence for its application in large lesions is limited. The present study therefore investigates the clinical and functional outcomes of AOT using a water-cooled diamond trephine system in patients with large osteochondral defects of the talus. We report one-year results regarding pain, ankle function, complications, and donor-site morbidity in a consecutive cohort treated with multi-plug resurfacing of the talar dome.

## 2. Materials and Methods

### 2.1. Study Design and Setting

This retrospective cohort study evaluated the clinical and functional outcomes of autologous osteochondral transplantation (AOT) for large, symptomatic osteochondral lesions of the talus with subchondral involvement, for which AOT was selected as a restorative procedure (typically after failure of non-operative treatment). All patients were treated between November 2009 and November 2014 at a tertiary orthopedic referral center in Hamburg, Germany. Data were anonymized and analyzed at the Department of Orthopedics and Traumatology, University Hospital Salzburg, Austria.

### 2.2. Patient Selection

Patients who underwent AOT between 2009 and 2014 using a standardized press-fit technique were eligible for inclusion if they were ≥14 years of age and presented with full-thickness osteochondral defects of the talar dome due to post-traumatic lesions or osteochondritis dissecans (OCD), classified as International Cartilage Repair Society (ICRS) grade III–IV. All patients underwent AOT using a standardized press-fit technique. For the present analysis, only patients with a minimum clinical follow-up of 12 months and sufficiently complete clinical documentation were included. Exclusion criteria were incomplete or failed surgical procedures, re-injury of the operated ankle during follow-up or incomplete/missing clinical data.

### 2.3. Data Collection

Clinical and operative data were obtained retrospectively from electronic health records, surgical protocols, and follow-up documentation. Standardized evaluations were scheduled at 3, 6, 9 and 12 months postoperatively, and clinical assessments were performed by the operating surgeon or a designated team member during routine follow-up. The surgical technique, rehabilitation protocol, and large parts of the methodological approach were identical to those previously described for knee AOT by Wittig-Draenert et al. [25], and sections of the present Methods description were adapted accordingly. Missing values were excluded from individual analyses. Because all operations and evaluations were performed by the same surgical team, inter-rater variability did not apply.

### 2.4. Surgical Technique

All procedures were performed by a single senior orthopedic surgeon with extensive experience in cartilage reconstruction, following a standardized operative protocol. Preoperative, patient-specific planning was conducted by the senior surgeon in all cases. Surgical intervention was indicated exclusively for patients with osteochondral defects classified as ICRS grade III or IV, based on preoperative imaging including magnetic resonance imaging (MRI) and plain radiographs.

**Figure 1:**
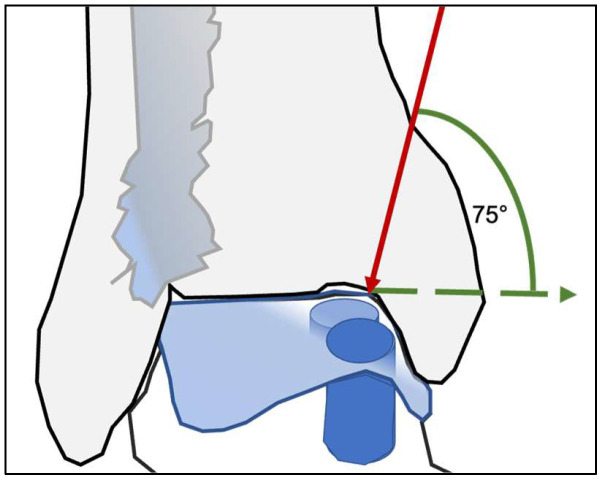
To achieve adequate access to the talar dome for osteochondral transplantation, a steep osteotomy of approximately 75° is required, performed either medially or laterally depending on lesion location.

Surgical access to the talar dome was determined by the location of the lesion. Medial lesions were approached through a medial malleolar osteotomy, whereas lateral lesions required an osteotomy of the distal fibula. To ensure adequate visualization of the talar dome and a safe working corridor, the osteotomy was performed with a minimum inclination of approximately 75°. This steep osteotomy angle allows controlled elevation of the malleolar fragment while preserving sufficient cortical stability for subsequent fixation (Figure 1).

Following medial malleolar osteotomy, fixation was standardized across cases using two cannulated screws; screw trajectories were predrilled prior to completing the osteotomy to facilitate accurate reduction and compression fixation at closure. No osteotomy-related complications were documented in this cohort. For the distal fibular osteotomy, a steep oblique cut was performed using an oscillating saw while preserving the syndesmosis where possible; when required, the anterior syndesmotic attachment at the Chaput tubercle was osteotomized en bloc and subsequently refixed. Reposition and fixation were performed using a standardized pre-plating technique with a one-third tubular plate (plate temporarily applied and holes predrilled, then removed for osteotomy and reapplied for definitive fixation). No fibular osteotomy–specific technical complications were recorded.

**Figure 2:**
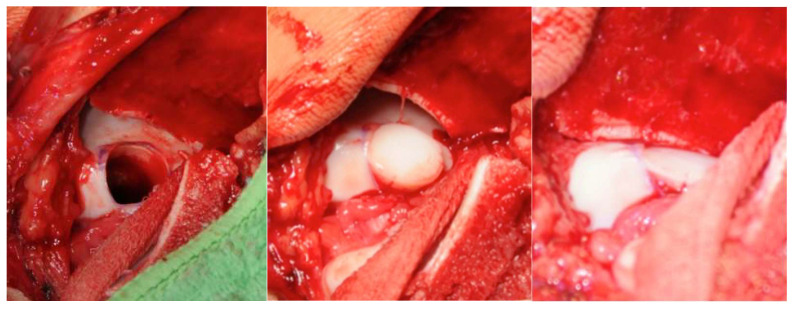
Intraoperative photographs illustrating autologous osteochondral transplantation of the talar dome. **Left**: prepared graft bed prior to implantation of osteochondral plugs. **Middle**: intraoperative appearance with both plugs positioned in situ, with one plug inserted and the second plug aligned prior to press-fit implantation. **Right**: final intraoperative result after press-fit implantation of both plugs, restoring the articular surface of the talar dome.

After exposure, the osteochondral defect was prepared using a water-cooled diamond trephine system. This technique permits precise, low-temperature removal of damaged cartilage and subchondral bone while minimizing thermal injury. Cylindrical osteochondral grafts (mean diameter 0.9 cm) were harvested from non–weight-bearing regions of the ipsilateral patellofemoral groove using the same diamond instrumentation to minimize thermal damage and receive highly congruent grafts. Graft depth was determined individually in regard to the defect geometry to achieve optimal primary stability; in most cases, plugs of up to approximately 18 mm were used, taking care to avoid violation of the sinus tarsi. Reconstruction of the talar dome was performed using a press-fit technique. Depending on lesion size and geometry, one to three overlapping grafts were inserted to recreate the native curvature of the talar articular surface. Attention was given to aligning the grafts flush with the surrounding cartilage and ensuring that subchondral bone contact was secure to promote early osseous integration (Figure 2).

Defect surface area (mm^2^) was derived retrospectively from operative records based on the number and standardized inner diameters of the cylindrical trephines used (A = π·r^2^), with estimated subtraction of overlapping cylinder areas; this represents a two-dimensional surface area measure, while the subchondral component was present in all cases but not quantified in the area calculation. Trephine size selection was performed intraoperatively by the operating surgeon based on visual assessment; formal measurement reliability testing was not performed.

All donor sites in the patellofemoral groove were reconstructed using autologous cylindrical bone plugs harvested from the iliac crest and covered with surrounding musculoskeletal tissue. The iliac crest harvest site was subsequently refilled with a press-fit hydroxyapatite substitute, and the musculotendinous layer was closed to reduce postoperative discomfort and support healing. Postoperatively, all patients followed a standardized rehabilitation protocol, including partial weight-bearing for up to six weeks. The rationale for this regimen was to protect the grafts during the early phase of osseous and chondral integration while allowing gradual restoration of ankle mobility.

### 2.5. Outcome Measures

Clinical outcomes were assessed using the following validated scores:

• Numeric Rating Scale (NRS) for pain;
• American Orthopaedic Foot & Ankle Society (AOFAS) Ankle–Hindfoot Score.

The NRS was recorded at routine follow-up visits, where patients rated their current pain on a scale from 0 (“no pain”) to 10 (“worst imaginable pain”). The AOFAS Ankle–Hindfoot Score was recorded at the same follow-up visits and evaluates ankle status across three domains: pain, function, and alignment. Scores range from 0 to 100, with higher values indicating better function.

Both scores were recorded preoperatively and at 3, 6, 9, and 12 months postoperatively. The primary endpoint was the change in pain and function over the 12-month follow-up period, while secondary endpoints included subgroup analyses according to graft configuration (mono-, double-, triple-cylinder chains), patient sex, age, and defect size. All examinations and score assessments were performed by the primary researcher, ensuring consistency in data collection.

### 2.6. Statistical Analysis

Data were analysed using generalized estimation equation models, whereby data were tested for normality. The Gaussian distribution was used as the underlying distribution and the identity function as the link function. The clustering unit was the patient ID, and time was used as within subject variable. Additionally, an independent working correlation structure as well as the robust estimator for the covariance matrix were used. Age and graft size were used as continuous covariates, time, graft location and sex as categorical factors. Main factors like age, tx site, tx size, time and sex were tested and corresponding LSD tests were done. 95% CI were computed and illustrated by using whisker plots. Spearman correlation coefficients and corresponding tests were also computed. Spearman correlation coefficients were computed for continuous variable associations. Confidence intervals (95% CI) were visualized using whisker plots. All analyses were conducted using IBM SPSS Statistics for Windows, Version 29.0.2.0 (IBM Corp., Armonk, NY, USA), and TIBCO Spotfire, Data Science Workbench Version 14. A *p*-value < 0.05 was considered statistically significant.

### 2.7. Quality Assessment

To reduce potential bias, data were extracted and verified by the principal investigator. Only complete clinical records with consistent follow-up were included in the final analysis. Because all surgeries were performed by a single high-volume surgeon, variability in graft placement was minimized. Complications were classified according to severity and relevance, including donor site morbidity, infection, bleeding, and nerve injury. No major intraoperative or post-operative complications occurred in the analyzed cohort.

### 2.8. Ethics

The study was approved by the Ethics Committee for the State of Salzburg (Austria). Given the retrospective and fully anonymized nature of the dataset, a waiver of informed consent was granted. All procedures adhered to the principles of the Declaration of Helsinki and national ethical standards. Patient confidentiality was ensured through strict data protection measures, including encrypted storage and limited access to authorized study personnel.

## 3. Results

This section presents the clinical characteristics and baseline data of the 40 patients who underwent autologous osteochondral transplantation (AOT) of the ankle. The patient cohort was derived from the same retrospective data collection that previously served as the basis for our analysis of autologous osteochondral transplantation of the knee [25], using an identical data extraction, documentation, and quality-control framework. The descriptive statistics summarize patient demographics, defect characteristics, and surgical details. These findings form the basis for subsequent evaluation of clinical outcomes over time.

### 3.1. Patient Demographics and Baseline Characteristics

During the study period, 49 patients met the clinical and intraoperative eligibility criteria. Nine patients were excluded from the final analysis due to loss to follow-up, resulting in missing outcome data: three patients had no postoperative clinical data recorded after surgery, five patients had no data beyond the 3-month follow-up visit, and one patient had no data beyond the 6-month follow-up visit.

The final study population thus consisted of 40 patients with a mean age of 32.18 years (range: 14–62 years, SD ± 13.7), and a median age of 31 years. Of these, 19 patients (47.5%) were female and 21 (52.5%) male. Laterality of the operated ankle was almost evenly split between right-sided (52.5%, *n* = 21) and left-sided (47.5%, *n* = 19) (Table 1).

Of the cohort, 33 patients (82.5%) had osteochondral lesions classified as ICRS grade III, whereas 7 patients (17.5%) demonstrated grade IV defects. Notably, all individuals with grade IV lesions were younger than 40 years, which may indicate that severe osteochondral damage tends to manifest earlier in this population and could correspond to the typical age of symptom onset.

### 3.2. Surgical Outcomes

The mean total grafted area was 137.4 (SD ± 31.9; range 71.63–225.39 mm^2^). A total of 35 transplants were placed on the medial talar dome, while 5 involved the lateral talus. Depending on defect size, reconstruction was achieved with one to three overlapping cylinders, with an average of 1.9 grafts per patient (33 patients received two grafts, 5 received a single graft, and 2 required three).

Graft harvesting was performed predominantly from the lateral patellar groove in 31 patients (77.5%), while the medial groove was used in 1 patient (2.5%), and both regions in 8 patients (20%). This variation reflected differences in the size and geometry of the talar defects.

No intraoperative complications occurred. Postoperatively, no patients experienced excessive bleeding, joint effusion, or prolonged donor-site discomfort. In addition, there were no infections, and none of the patients required conversion to ankle arthroplasty or arthrodesis within the follow-up period. The absence of notable donor-site morbidity supports the reproducibility and safety of the described technique.

### 3.3. Pain Reduction over Time (NRS for Pain)

Pain levels were evaluated using the NRS for pain before surgery and at 3, 6, 9, and 12 months postoperatively. Preoperatively, the mean NRS across all patients (*n* = 40) was 5.69 (SD ± 2.52), reflecting moderate to severe pain. Within three months after the procedure, pain scores dropped markedly to an average of 2.18 (SD ± 2.04). Further improvements were recorded at 6 months (mean 1.19, SD ± 1.35) and 9 months (mean 1.04, SD ± 1.23), with the lowest values seen at the one-year follow-up (mean 0.53, SD ± 0.98).

**Figure 3:**
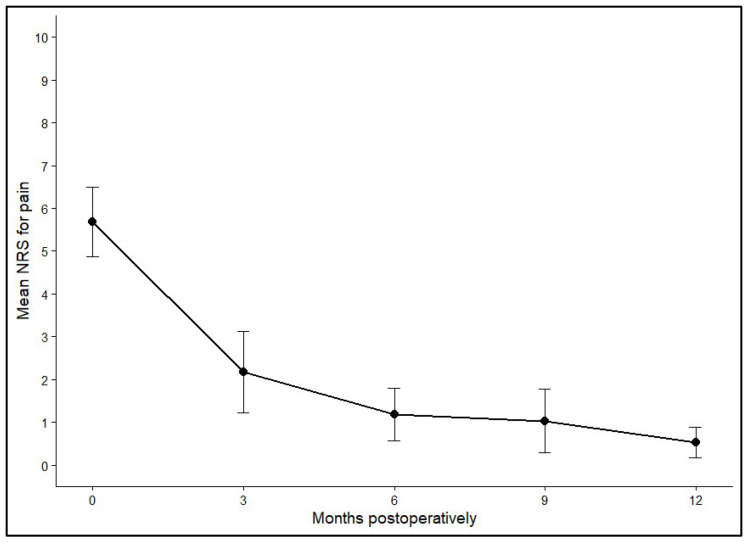
Mean NRS with 95% confidence intervals at baseline (0), 3, 6, 9, and 12+ months postoperatively.

**Figure 4:**
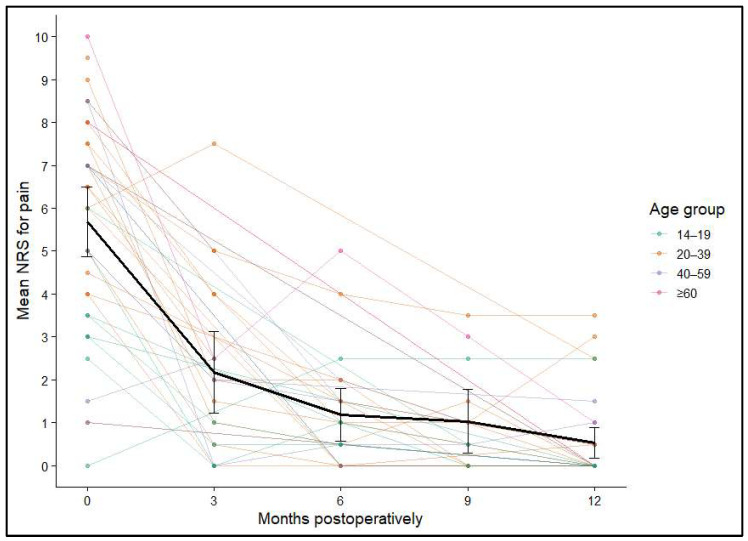
Individual NRS trajectories over 12 months, stratified by age group; mean and 95% confidence intervals shown in black.

Statistical analysis using Generalized Estimating Equations (GEEs) demonstrated a significant temporal effect on pain reduction (*p* < 0.001). Pairwise comparisons confirmed that pain levels at every postoperative timepoint were significantly lower than before surgery (all *p* < 0.001). Additional improvements were evident between 3 and 6 months (*p* = 0.029), 3 and 9 months (*p* = 0.021), 3 and 12 months (*p* < 0.001), and between 6 and 12 months (*p* = 0.012). By contrast, no meaningful differences were observed between the 6- and 9-month or the 9- and 12-month assessments, indicating that most pain relief occurred within the first half-year and then remained stable. Figure 3 and Figure 4 illustrate the average NRS score and its 95% confidence interval across these times post-operatively.

In summary, the most notable decline in pain occurred during the first 3 months following surgery, with sustained low levels throughout the remainder of the observation period (*p* < 0.001). Multivariable modeling revealed that graft location (*p* = 0.006) and patient age (*p* = 0.025) were significant predictors of postoperative pain, whereas graft size (*p* = 0.808) and sex (*p* = 0.179) showed no significant associations.

### 3.4. Functional Outcomes: AOFAS Ankle–Hindfoot Score

**Figure 5:**
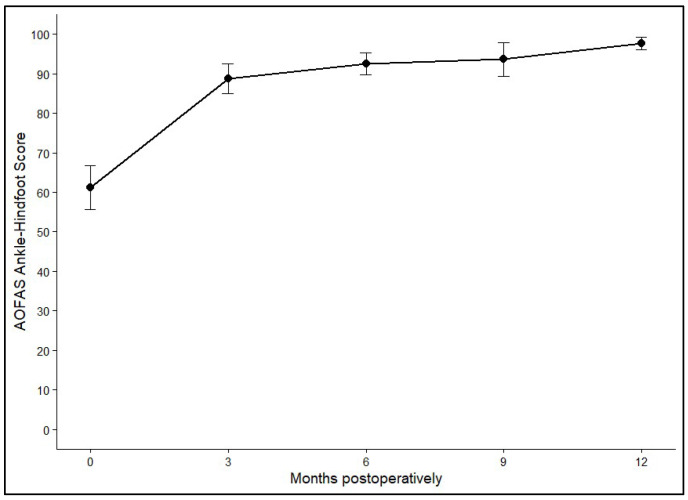
Mean AOFAS Ankle–Hindfoot Scores at baseline and at 3, 6, 9, and 12+ months postoperatively. Error bars represent 95% confidence intervals.

**Figure 6:**
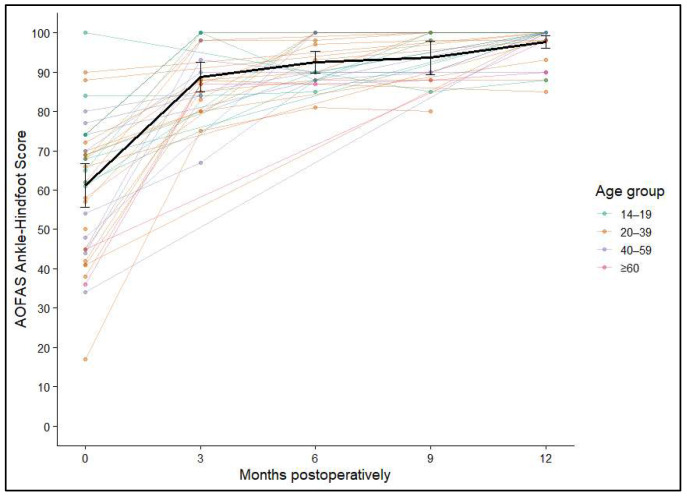
Individual AOFAS Ankle–Hindfoot Score trajectories over 12 months, stratified by age group; mean and 95% confidence intervals shown in black.

Postoperative ankle function was evaluated using the AOFAS Ankle–Hindfoot Score, which showed clear and durable improvement after AOT. The mean preoperative score of 63.79 (SD ± 2.55) rose sharply to 92.01 (SD ± 2.43) within the first three months. Further gradual increases were observed at 6 months (94.89, SD ± 2.76) and 9 months (96.13, SD ± 2.89), with values reaching 97.36 (SD ± 2.49) at 12 months or later. Statistical analysis confirmed a highly significant effect of time on functional outcome (*p* < 0.001). The greatest improvement occurred during the early postoperative phase, while functional scores remained consistently high and stable after six months. Figure 5 and Figure 6 show the average AOFAS score and its 95% confidence interval.

No significant associations were identified between ankle function and either sex (*p* = 0.245) or graft size (*p* = 0.97). In contrast, both patient age and the anatomical site of transplantation suggested a potential association (*p* = 0.052) but did not reach statistical significance. Overall, the AOFAS Ankle–Hindfoot Score findings indicate robust and lasting functional recovery after AOT, with the most pronounced gains occurring in the early postoperative period and maintained throughout long-term follow-up.

## 4. Discussion

This study addressed an important clinical gap in the treatment of large osteochondral lesions of the talus (OLT), a defect category for which bone marrow stimulation is widely considered insufficient and evidence for autologous osteochondral transplantation (AOT) remains limited. The clinical outcomes of multi-plug AOT performed with a water-cooled diamond trephine system were evaluated in patients with lesions averaging 140 mm^2^. Current reviews and consensus statements recommend osteochondral transplantation or osteo-periosteal grafting for lesions larger than approximately 100 mm^2^ or 10 mm in diameter, for cystic defects, and for cases after failed primary surgery [5,6,16,17]. In this context, one-year outcomes from the patient cohort of this study indicate that multi-plug AOT can reliably restore function even when a substantial portion of the talar dome has to be resurfaced.

A marked and durable reduction in pain and a substantial improvement in ankle function could be observed within the first postoperative year. These magnitudes are comparable to, or slightly better than, those reported in larger series and meta-analyses of AOT for OLT, where mean NRS reductions of about 4 points and improvements of approximately 25–30 points in commonly used ankle outcome scores are typical [22]. The greatest clinical change in our cohort occurred within the first 3–6 months and then plateaued, which aligns with other reports describing early gains followed by stable mid-term results after osteochondral transplantation of the talus [26,27].

Lesion size itself was not associated with worse pain or functional outcomes in our analyses, whereas older age and graft location showed a significant relationship with postoperative pain. Similar patterns have been described in several other studies, which identified age, BMI, lesion depth, cystic change, and containment type as more consistent predictors of outcome than area alone [1,28,29]. Our findings support the notion that once a certain critical size threshold is exceeded and the talar dome is reconstructed with osteochondral plugs, patient-related factors like age and BMI and local biomechanics may be more relevant for prognosis than the exact defect size.

Access to medial lesions in this patient cohort required a medial malleolar osteotomy, which is consistent with large systematic reviews reporting that the majority of OLTs are medial and that osteotomy is commonly necessary for perpendicular access during AOT or mosaicplasty [5,22]. A water-cooled diamond trephine system was used to harvest and implant relatively deep plugs (up to ~18 mm) in a press-fit fashion, aiming to maximize subchondral contact and primary stability. Experimental work has emphasized the importance of restoring contact area and pressure distribution for successful osteochondral reconstruction, and has shown that cylindrical press-fit grafts can normalize joint loading when congruency is achieved [12,30,31]. Histologic studies suggest that osseous integration of transplanted plugs occurs within 4–6 weeks, with revascularization of the subchondral plate during this period [32,33]. Our rehabilitation protocol—partial weight-bearing for six weeks followed by gradual loading—is in line with this biological timeframe and with commonly used regimens reported in contemporary series [34,35].

A central concern with knee-to-ankle AOT is donor-site morbidity at the patellofemoral joint. Meta-analyses and systematic reviews report knee donor-site pain rates of roughly 6.7–16.9%, with higher rates in smaller series and when three or more plugs are harvested [22,36,37]. Recent work on osteo-periosteal grafts from the iliac crest or tibia suggests promising results while avoiding knee donor morbidity, but long-term data remain sparse and most series are small [5,6,38]. While no knee-specific score for donor-site pain was assessed during the investigation, none of our patients reported persistent donor-knee symptoms at the latest follow-up. This favorable donor-site profile may be related to careful reconstruction of the harvest site using cylindrical iliac crest bone plugs, coverage with the local soft-tissue layer—including fibers that anchor to bone through Sharpey’s fibers—and refilling of the iliac crest with a press-fit hydroxyapatite substitute. This method conceptually reflects experimental work showing effective osteochondral repair with periosteum-covered bone grafts [32,38]. Nevertheless, given the relatively small cohort and the absence of standardized knee-specific outcome scores or imaging, our data should be interpreted cautiously; subtle donor-site changes may have been missed.

This study has several limitations. First, its retrospective design and lack of a control group limit direct comparison with alternative treatments, such as bone marrow stimulation, osteo-periosteal grafting, or osteochondral allografts. Second, all surgeries were performed by a single high-volume surgeon in a specialized center; while this reduces technical variability, it limits generalizability. Third, lesion size and graft con-figuration were heterogeneous, and postoperative imaging was not routinely obtained, preventing assessment of graft incorporation or cartilage congruency. Finally, the follow-up period of one year is insufficient to characterize long-term survivorship or the risk of late degenerative changes. Despite these limitations, our results provide consistent evidence that multi-plug AOT can be effective for large OLT.

AOT remains a well-established option for larger or complex talar osteochondral lesions, but it is increasingly relevant to benchmark its clinical gains against newer biomimetic/synthetic osteochondral scaffold constructs that aim to restore the osteochondral unit while avoiding donor-site morbidity [39,40]. Future research should therefore include randomized or well-matched comparative studies, comparing multi-plug AOT with other restorative options for large OLT, including osteo-periosteal grafts, matrix-augmented BMS, and fresh allografts, with standardized imaging and long-term follow-up to investigate whether the favorable outcomes observed in this and similar series translate into superior joint preservation and lower rates of arthrodesis or arthroplasty over decades. Finally, detailed evaluation of donor-site reconstruction strategies, including randomized assessment of re-filled versus non-refilled knee harvest sites, is needed to determine the optimal balance between talar restoration and donor morbidity.

## 5. Conclusions

In this retrospective cohort of patients with large, full-thickness osteochondral lesions of the talus, multi-plug autologous osteochondral transplantation using a water-cooled diamond trephine system resulted in substantial and durable improvements in pain and ankle function during the first postoperative year. No relevant complications or persistent donor-site morbidity at the knee were detected. This low donor-site morbidity further suggests that careful donor-site reconstruction, given the same priority as the talar defect, may contribute to favorable overall outcomes. These findings support AOT as an effective joint-preserving option for extensive OLT when less invasive procedures are likely to be insufficient.

While the single-center design and limited follow-up period restrict generalizability, the consistency of clinical improvement across lesion sizes and patient subgroups indicates that multi-plug AOT may be a robust reconstructive strategy for extensive OLT. Prospective, comparative studies with long-term structural and functional outcomes are needed to clarify its role within the broader treatment algorithm and to determine whether the favorable one-year results observed here translate into sustained joint preservation.

## Figures and Tables

**Table 1:** Demographic and clinical characteristics by age group.

Category	Total (*n* = 40)	14–24 Years	25–39 Years	40–59 Years	60–74 Years
**Sex, *n* (%):**					
- Female	19 (47.5%)	9	6	4	0
- Male	21 (52.5%)	5	8	6	2
**Side of Surgery, *n* (%):**					
- Right Ankle	21 (52.5%)	8	9	3	1
- Left Ankle	19 (47.5%)	6	5	7	1
**ICRS Grade, *n* (%):**					
- Grade III	33 (82.5%)	11	10	10	2
- Grade IV	7 (17.5%)	3	4	0	0
**Total Graft Size (mm^2^):**					
- Mean (SD)	137.4 (31.9)	144.9 (21.4)	142.0 (40.4)	125.2 (24.3)	114.1 (60.1)
- Range	71.6–200.8	99.9–172.8	88.3–200.8	103.1–172.8	71.6–156.6

## Data Availability

The data presented in this study are available upon reasonable request from the corresponding author due to privacy restrictions.

## Abbreviations

The following abbreviations are used in this manuscript:

AOFAS	American Orthopaedic Foot & Ankle Society
AOT	Autologous Osteochondral Transplantation
CI	Confidence Interval
GEE	Generalized Estimating Equation
ICRS	International Cartilage Repair Society
LSD	Least Significant Difference
NRS	Numeric Rating Scale
OLT	Osteochondral Lesion of the Talus
SD	Standard Deviation
SPSS	Statistical Package for the Social Sciences
